# The Landscape and Regulatory Determinants of A-to-I RNA Editing in *Escherichia coli* and *Pseudomonas aeruginosa* Isolated From Patients With Urinary Tract and Ear Infections

**DOI:** 10.1093/infdis/jiaf645

**Published:** 2025-12-24

**Authors:** Eyal Elias, Danielle Keidar-Friedman, Nadav Sorek, Orit Raz, Sharon Ovnat Tamir, Liam Aspit, Dan Bar Yaacov

**Affiliations:** The Shraga Segal Department of Microbiology, Immunology, and Genetics, Ben-Gurion University of the Negev, Beer Sheva, Israel; Assuta Ashdod University Hospital, Faculty of Health Sciences, Ben Gurion University of the Negev, Ashdod, Israel; Assuta Ashdod University Hospital, Faculty of Health Sciences, Ben Gurion University of the Negev, Ashdod, Israel; Assuta Ashdod University Hospital, Faculty of Health Sciences, Ben Gurion University of the Negev, Ashdod, Israel; Assuta Ashdod University Hospital, Faculty of Health Sciences, Ben Gurion University of the Negev, Ashdod, Israel; The Shraga Segal Department of Microbiology, Immunology, and Genetics, Ben-Gurion University of the Negev, Beer Sheva, Israel; The Shraga Segal Department of Microbiology, Immunology, and Genetics, Ben-Gurion University of the Negev, Beer Sheva, Israel

**Keywords:** A-to-I RNA editing, bacteria, hospitalized patients, *Escherichia coli*, *Pseudomonas aeruginosa*

## Abstract

**Background:**

Adenosine-to-inosine (A-to-I) mRNA editing can alter protein sequence and function, enabling bacteria to express two RNA and protein versions encoded by the same gene. However, its prevalence and significance in clinical bacterial settings remain unclear.

**Methods:**

We collected ten *Escherichia coli* and seven *Pseudomonas aeruginosa* isolates from hospitalized patients with urinary tract infections (UTI) or ear infections. Whole-genome and transcriptome sequencing were performed for each isolate, followed by Sanger sequencing for selected sites.

**Results:**

We present the first comprehensive analysis of A-to-I RNA editing in pathogenic bacteria isolated from hospitalized patients. We identified dozens of A-to-I RNA editing sites, including novel sites not previously reported in nonpathogenic *E. coli* and *P. aeruginosa* strains. We found that *E. coli* exhibits higher editing levels and a greater number of editing sites than *P. aeruginosa*. Most editing sites are embedded within a conserved 7-base motif and are frequently located in predicted stem-loop RNA secondary structures, highlighting the importance of both sequence and structure for editing site recognition in both the examined species. Most editing events occur in mRNA and often result in nonsynonymous amino acid changes, with a notable prevalence of tyrosine-to-cysteine substitutions. Finally, we observed that editing patterns are similar between antibiotic-resistant and sensitive isolates, suggesting a more general role in the biology of the examined species.

**Conclusions:**

Adenosine-to-inosine RNA editing is a feature of pathogenic bacteria isolated from clinical samples. Our findings expand current knowledge of bacterial RNA editing in clinical contexts and provide a framework for future functional investigations.

## Teaser

Here, we describe the occurrence, regulatory features, and predicted effect of RNA-specific sequence changes resulting from A-to-I RNA editing in *E. coli* and *P. aeruginosa* isolates obtained from hospitalized patients.

Adenosine-to-inosine (A-to-I) RNA editing, a post-transcriptional modification, occurs when adenosine is deaminated to inosine [1, 2], which during translation or reverse transcription is read as guanosine [3]. Thus, when A-to-I RNA editing occurs within open reading frames, it could change protein sequence [2], and affect the structure and activity of different proteins [4]. A-to-I RNA editing was shown to be important in different organisms, for example, by preventing auto-immunity and neurological impairments [2, 5–14].

A-to-I RNA editing in prokaryotes is far less explored compared to eukaryotes. The editing enzyme in bacteria, tRNA-specific adenosine deaminase (TadA), was shown to be essential in bacteria, and deaminates adenosine 34 at the wobble position of *tRNA-Arg2* [15–17]. In contrast, A-to-I mRNA editing was only recently discovered in bacteria, with TadA as the mediating enzyme [17–20]. Adenosine-to-inosine mRNA editing was found to occur in genes that are important for response to infection-relevant stimuli, bacterial persistency, and pathogenicity [17–20]. Recently, we showed that A-to-I mRNA editing is found in 64 species and constitutes a novel mechanism to introduce protein isoforms with altered function in bacteria [17, 21]. Despite recent advancements in our understanding of bacterial RNA editing, to the best of our knowledge, A-to-I mRNA editing has never been explored in pathogenic bacteria from clinical samples.

Urinary tract infection (UTI) is one of the most common types of infections [22]. *Escherichia coli* is the most common cause of community-acquired and nosocomial UTI, leading to substantial medical costs, morbidity and mortality worldwide [23–25]. In some cases, the *E. coli* strains show a concerning antimicrobial resistance (AMR), which leads to even more complications [26]. This alarming trend demands further research in the field to inquire more about the genes and other factors that provide bacterial AMR.

Another common infection is ear infection (otitis) with millions of cases globally [27], particularly in children [28–33]. Many cases of otitis externa are caused by *Pseudomonas aeruginosa* [34], which causes both community-acquired and nosocomial infections [35]. Some *P. aeruginosa* strains exhibit high levels of AMR to a broad spectrum of antibiotics [36], making them difficult to treat. Thus, it is important to gain a better understanding of *P. aeruginosa* mechanisms of infection and resistance to antibiotic treatments.

Here, we focus on identifying the landscape and regulatory determinants of A-to-I mRNA editing in clinical isolates of *E. coli* from UTI samples, and *P. aeruginosa* from ear infections, and examining its correlation with patient and isolate clinical parameters.

## METHODS

### Isolation of Bacteria From Clinical Urine and Ear Infection Samples

Clinical samples were taken from patients admitted to Assuta Ashdod University Hospital.

Urine samples were taken from different locations of the urinary tract and catheters. Urine samples were delivered to the clinical microbiology laboratory and plated on tryptic soy agar (TSA) sheep blood (5%) and CHROMagar Orientation agar plates (Hylabs, Rehovot, Israel) within 2 hours after receiving the samples, using a 1 or 10 µL bacteriological loop needle. Plates were incubated at 35 ± 2°C for 24–48 hours.

Ear infection samples were taken from the patients’ middle or outer ear using a Transystem™ cotton swab (Copan Diagnostics, Murrieta, CA). Swab samples were delivered to the clinical microbiology laboratory and plated according to protocol. The plates were incubated at 35 ± 2°C and 5% CO_2_ for 24–96 hours.

Bacterial colonies from the positive urine and ear cultures were identified using Matrix-assisted laser desorption/ionization-time of flight mass spectrometry (MALDI-TOF MS, bioMérieux, Marcy-l'Étoile, France) analysis. Pure urine or ear cultures of *E. coli* or *P. aeruginosa*, respectively, were collected, and the bacteria were isolated on MacConkey agar plates (Hylabs, Rehovot, Israel). A single colony was selected from the isolated *E. coli* and was inoculated into a stock tube of BHI broth + glycerol (Hylabs, Rehovot, Israel) and stored at −80°C.

Antibiotic susceptibility testing was done using VITEK® MS (bioMérieux, Marcy-l'Étoile, France). An antibiogram was made for each bacterial isolate, and isolates that were found to be resistant or susceptible to certain antibiotics (see Supplementary Table 5) were selected for the research.

### DNA and RNA Extractions

Each bacterial isolate was cultured in BHI (hylabs #TT244/30) broth media. First, the bacteria were plated from the −80°C stock to 3 mL of BHI media in each well of the culture plate (Costar®, Corning, New-York, USA-6 well) for an overnight culture incubated at 37°C with 200 rpm for 24 hours in Heidolph® plate incubator and shaker. The overnight culture was then diluted (1:100) to a fresh 6 mL of BHI broth and grown for 2 hours (*E. coli*) and 3–4 hours and 20 minutes (*P. aeruginosa*) till the bacterial cultures reached their mid-logarithmic phase (at 2.5–4 McFarland). Of this, two tubes of 1.5 mL were centrifuged for 2 minutes at 13 000 rpm, the supernatant was removed, and the cell pellet was used for DNA and RNA extraction. DNA was extracted using GeneJET Genomic DNA Purification Kit (Thermo Scientific #K0721) according to the manufacturer protocol. RNA was extracted using GeneJET RNA Purification Kit (Thermo Scientific #K0731) according to the manufacturer protocol. RNA samples were treated with 4 units of DNase I (NEB # M0303L) for 20 minutes at 37°C and cleaned using RNA Clean & Concentrator™ (Zymo ZR-R1015).

### DNA-seq and RNA-seq

Ribosomal RNA was depleted using NEBNext® rRNA Depletion Kit (Bacteria) (New England Biolabs, # E7850). Libraries were constructed using NEBNext® Ultra™ II Directional RNA Library Prep Kit for Illumina® (New England Biolabs, #E7760). DNA-seq libraries were prepared using NEBNext® Ultra™ II FS DNA Library Prep Kit for Illumina (New England Biolabs, #E7805L). Finally, RNA-seq and DNA-seq libraries were sequenced on the NovaSeq X platform (Illumina).

### Bioinformatic Analysis of RNA Editing Sites

We used CLC Genomics Workbench for all analysis steps. In brief, RNA-seq reads were first trimmed according to length and quality scores, and paired reads were merged into a single longer read, maintaining the orientation of the R1 reads.

Next, for the *E. coli* and *P. aeruginosa* RNA-seq and DNA-seq reads, the reads were mapped to the uropathogenic *E. coli* (UPEC) CFT073 reference genome and the *P. aeruginosa* UCBPP-PA14 reference genome, respectively.

RNA variant calling was performed and filtered to retain variants that match all the following criteria: Frequency (editing level) ≥1%; Unique reads supporting a variant ≥3; Read coverage ≥10.

Next, we removed the variants that were present in the DNA control reads.

Finally, using the nonredundant list of putative editing events, we extracted the state of each site harboring an editing event from every sample (isolate). At the end of this process, for an editing event to be considered, it has to have a coverage of at least 4 reads, at least 2 reads supporting an event, with editing level above 1%, and present in at least 2 samples.

### RNA Editing Analysis of *E. coli* CFT073 and *P. aeruginosa* UCBPP-PA14 Reference Strains

We analyzed published datasets [37, 38] where bacteria were untreated and grown under conditions closely matching ours, including rich media (LB or cation-adjusted Mueller–Hinton), and mid-log phase harvesting (optical density [OD] 0.4–0.6).

### cDNA Preparation, PCR Amplification and RNA Editing Validation Using Sanger Sequencing

Following incubation of RNA extraction for 15 minutes at 65°C, cDNA synthesis was performed using GoScript Reverse Transcription Mix (Promega # A2801), following the manufacturer's protocol. The flanking sequence of each editing site was amplified using PCR with specific primers for each site and specific annealing temperature (Supplementary Table 9). The samples were cleaned using the Zymo DNA Clean & Concentrator™ kit (#D4004), and sent for Sanger sequencing.

### Sequence Motif and Secondary Structure Prediction Around Editing Events

We used WebLogo to identify the sequence motif around editing events [39]. We used RNAfold from the ViennaRNA Package 2.0 to calculate the minimum free energy (MFE) around editing events. We calculated the structure of the MFE of 17 nucleotides around edited sites (which is the length of *tRNA-Arg2* anticodon arm). We position the edited adenosine as the “0” position in our sliding window analysis similar to its location in the anticodon arm of *tRNA-Arg2*.

### Statistical Analysis

We used PRISM 10 or Excel to conduct the statistical analyses described in the text.

## RESULTS

### The Landscape of Adenosine-to-inosine RNA Editing in Urinary Tract Infection *E. coli* Isolates

**Figure 1.**
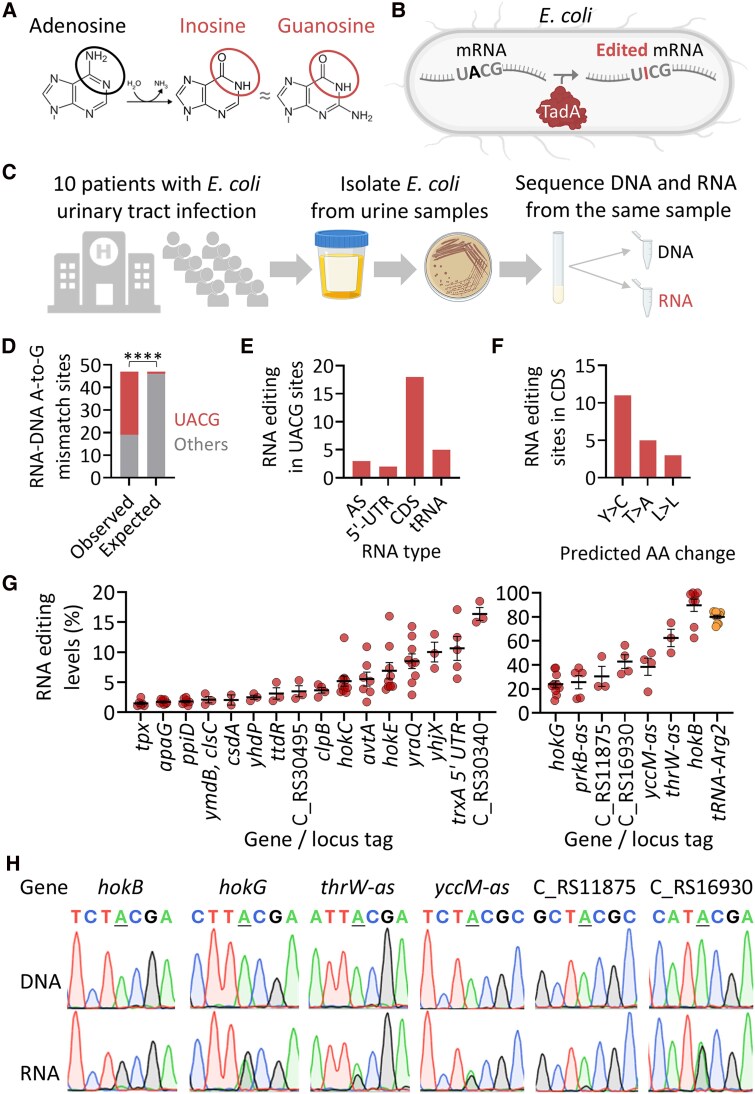
The landscape and levels of A-to-I RNA editing in uropathogenic *E. coli* isolated from patients. *A*, Adenosine is deaminated into inosine, which is recognized by the ribosome and reverse transcriptase as guanosine, allowing its detection in sequencing data. *B*, TadA can edit mRNAs containing a four-base sequence motif of UACG (TACG at the DNA level). *C*, Experimental design: *E. coli* isolated from 10 patients with UTI was grown at 37°C in Brain-Heart infusion broth (BHI). RNA and DNA were extracted from the same sample at mid-logarithmic phase and RNA-seq and DNA-seq were performed. *D*, RNA-DNA A-to-G mismatches are significantly enriched in the UACG motif (Fisher's exact test; *P* < .0001). *E*, Distribution of editing events according to RNA type. Abbreviations: AS, antisense; UTR, untranslated region; CDS, coding sequence; tRNA, *tRNA-Arg2*. *F*, predicted amino acid (AA) change by RNA editing when in CDS. *G*, an overview of RNA editing levels in identified transcripts. On the left panel are sites with an average editing of below 20%. On the right panel are sites with an average editing of above 20%. Only sites that passed our filters in at least two samples are displayed (see Methods). In unannotated genes, the locus tag is mentioned. Each editing event shown here must have a coverage of at least 4 reads, at least 2 reads supporting an event, with editing level above 1% (fraction of RNA reads with A-to-G mismatch, that are not found in the DNA data), and present in at least 2 samples (isolates). *H*, Sanger sequencing validates the occurrence of editing events in corresponding DNA and RNA samples. The edited “A” is centered and underlined, and the editing signal is represented by a guanosine (black) peak in the RNA samples. The genomic positions of the edited sites are found in Supplementary Table 1.

To characterize the A-to-I RNA editing landscape in *Escherichia coli* isolated from patients with UTIs, we performed parallel RNA and DNA sequencing on 10 clinical isolates. Candidate A-to-I editing sites were identified as A-to-G RNA-DNA mismatches (Figure 1*A*–*C*). Sequence analysis of regions flanking these mismatches revealed significant enrichment for the UACG motif (positions “−1” to “+2” relative to the edited “A”), the same sequence recognized by TadA as essential for its editing activity (Figure 1*D*, Supplementary Figure 1A, Supplementary Table 1). The majority of identified UACG motif-containing sites were located within coding sequences (CDS), with additional sites mapped to intergenic regions and antisense (AS) RNAs (Figure 1*E* and Supplementary Table 1).

We also detected the canonical TadA-mediated editing event at position A34 of *tRNA-Arg2*, transcribed from five distinct gene loci (Figure 1*E* and Supplementary Table 1). Importantly, most editing events within CDS were predicted to alter the corresponding protein sequence (Figure 1*F* and Supplementary Table 1).

In total, we identified 23 UACG-embedded A-to-I RNA editing sites in addition to the canonical *tRNA-Arg2* site (Figure 1*G* and Supplementary Table 1). Of these, 15 sites—including loci such as *hokG* and the unnamed gene in locus tag “C_RS16930”—are novel and were not previously reported. The remaining 8 sites had been previously described in nonpathogenic *E. coli* strains [18]. Editing levels across transcripts varied widely, ranging from 1.48% in *tpx* to 89.59% in *hokB* (Figure 1*G* and Supplementary Table 1). Notably, most editing events were detected in only a subset of clinical isolates, indicating variability in editing patterns among pathogenic *E. coli* strains. Consistent with findings in laboratory strains, several *hok* transcripts were edited, with *hokB* exhibiting the highest editing levels—surpassing even the canonical *tRNA-Arg2* site. Interestingly, RNA-seq experiments from the *E. coli* CFT073 laboratory strain grown under similar conditions revealed editing at only 10 of the 23 sites identified in our clinical isolates (Supplementary Figure 1B and Supplementary Table 2), indicating possible functional divergence.

To validate our computationally identified editing events, we performed Sanger sequencing of both DNA and RNA from corresponding samples for six sites with average editing levels above 20% (Figure 1*G* and Supplementary Table 1). In all cases, the edited allele was confirmed at the RNA level, providing further experimental validation of our NGS-based approach (Figure 1*H*).

Taken together, these analyses identified the repertoire of *bona fide* A-to-I RNA editing sites in clinical uropathogenic *E. coli* isolates, including multiple novel events, and highlight the diversity of editing among strains from human patients.

### Adenosine-to-inosine RNA Editing Occurs at Low Levels in Seven mRNAs of *P. aeruginosa* Isolated From Patients

**Figure 2.**
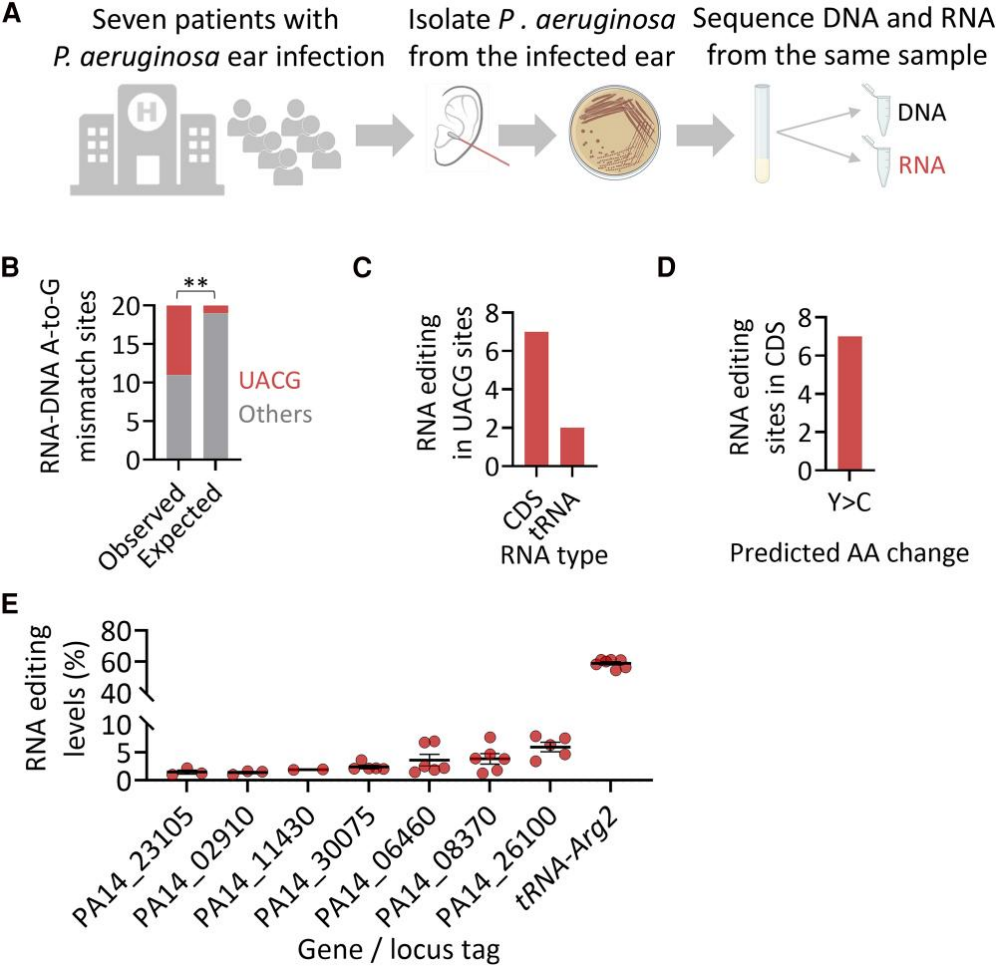
Adenosine-to-inosine RNA editing occurs at low levels in seven transcripts in *P. aeruginosa* isolated from patients with ear infection. *A*, Experimental design: *P. aeruginosa* isolated from seven patients with ear infection was grown at 37°C in BHI broth. RNA and DNA were extracted from the same sample at mid-logarithmic phase and RNA-seq and DNA-seq were performed. *B*, RNA-DNA A-to-G mismatches are significantly enriched in the UACG motif (Fisher's exact test; *P* < .01). *C*, Distribution of editing events according to RNA type. *D*, Predicted amino acid (AA) change by RNA editing events in CDS. *E*, An overview of RNA editing levels in identified transcripts. In unannotated genes, the locus tag is mentioned. Each editing event shown here has to have a coverage of at least 4 reads, at least 2 reads supporting an event, with editing level above 1% (fraction of RNA reads with A-to-G mismatch, that are not found in the DNA data), and present in at least 2 samples (isolates).

Next, we aimed to determine the A-to-I mRNA editing landscape in *P. aeruginosa* isolated from patients with ear infections in which it was the sole causative agent. To this end, we sequenced RNA and DNA from seven *P. aeruginosa* isolates to identify A-to-G RNA-DNA mismatches that could represent A-to-I editing events (Figure 2*A*). Analysis of the sequence context surrounding these mismatches revealed a significant enrichment for the UACG motif, consistent with the recognition motif of TadA (Figure 2*B*, Supplementary Figure 2A and Supplementary Table 3).

As in the case of uropathogenic *E. coli*, we detected the canonical editing event at A34 on *tRNA-Arg2*, here transcribed from two different genes (Figure 2*C* and Supplementary Table 3). All other UACG sites (n = 7) are novel sites that occur in CDS and are predicted to alter protein sequence (Figure 2*C*-*D* and Supplementary Table 3). Analyzing RNA-seq data from laboratory *P. aeruginosa* (UCBPP-PA14) grown to mid-log phase in rich medium detected editing at only 4 of the 8 sites seen in clinical isolates (Supplementary Figure 2B and Supplementary Table 4), again suggesting functional differences between strains.

Importantly, all mRNA editing events (found in CDS) in the *P. aeruginosa* isolates were found in low levels (<8%) (Figure 2*E*). Thus, compared to uropathogenic *E. coli*, A-to-I mRNA editing in pathogenic *P. aeruginosa* is less abundant and is found in lower levels under our culture conditions.

### Adenosine-to-inosine mRNA Editing Events in *E. coli* and *P. aeruginosa* are Predominantly Localized to a Conserved Seven-base Motif Within Stem-loop Structures

Our previous analysis across 64 gammaproteobacterial species revealed that most A-to-I mRNA editing events occur within a conserved 7-base motif (YUACGAA; positions “−2” to “+4” relative to the edited “A”) [17]. However, given the broad phylogenetic range analyzed earlier (381 sites), potential species-specific sequence preferences may have been masked. To address this, we examined the sequence context surrounding A-to-I mRNA editing sites specifically in *E. coli* and *P. aeruginosa*.

**Figure 3.**
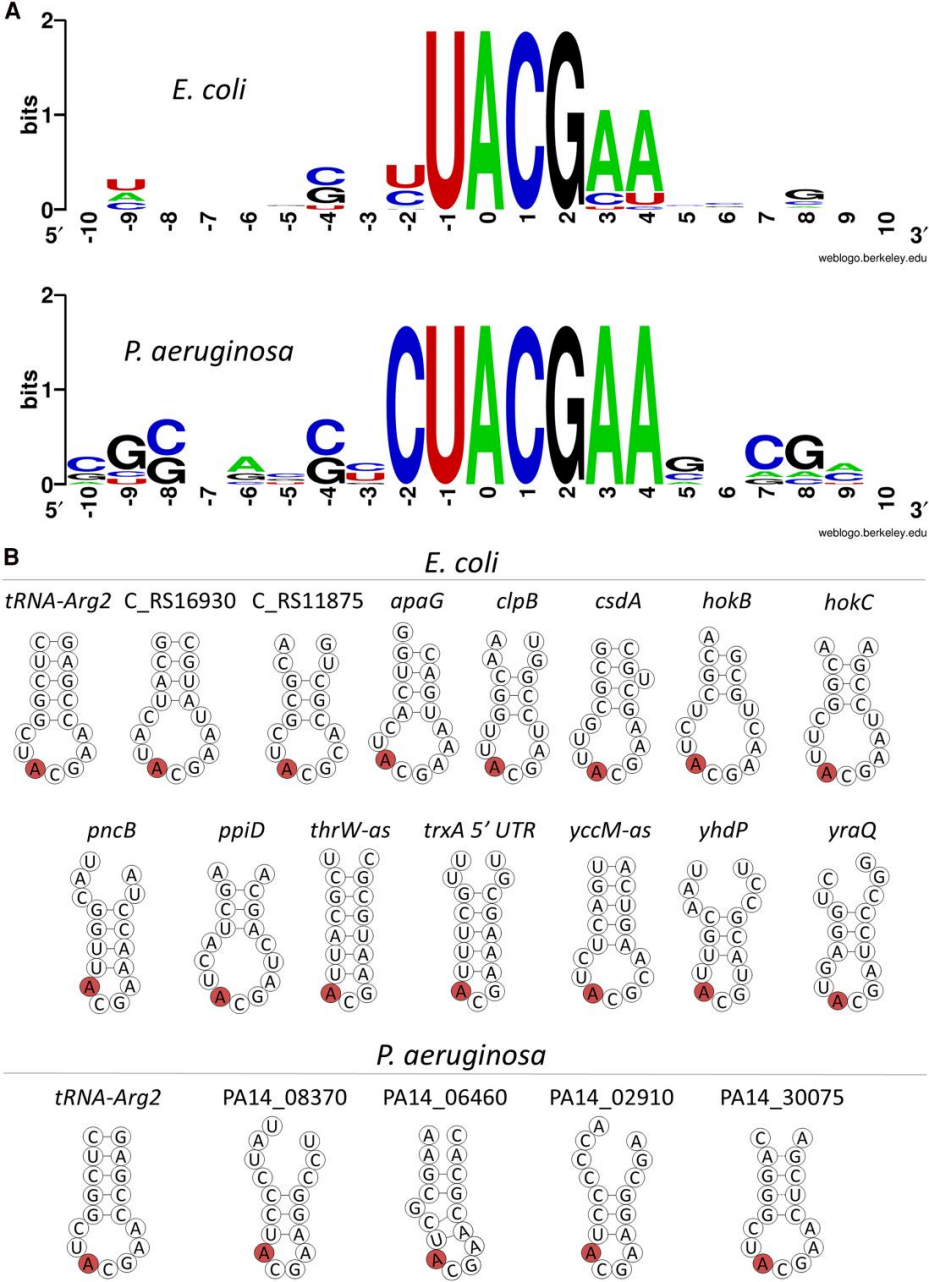
Adenosine-to-inosine mRNA editing events are mostly found in a seven-base motif embedded within a stem-loop structure. *A*, WebLogo analysis of the 23 mRNA editing events detected in *E. coli* (Top) and seven mRNA editing events detected in *P. aeruginosa* (bottom). Position “0” is the edited site. *B*, Minimum free energy (MFE) secondary structure predicted by RNAfold around the A-to-I editing site (red) for the 17 nucleotides composing the anticodon arm of *tRNA-Arg2*, and for the 17 nucleotides around 14 mRNA edited sites in *E. coli* and four mRNA edited sites in *P. aeruginosa*. In unannotated genes the locus tag is mentioned. In *E. coli* it is preceded by “C_RS” and in *P. aeruginosa* by “PA14_.” It is not included in the figure itself due to limitation of space All structural predictions are found in Supplementary Figures 3–5.

In *E. coli*, we confirmed enrichment of the same 7-base motif as previously reported [17] (Figure 3*A*). Notably, we also identified a certain degree of conservation at position “–4” (four bases upstream of the edited adenosine), with either a “G” or “C” consistently present—an observation not highlighted in prior analyses. Similarly, all editing events in *P. aeruginosa* also conformed to this 7-base motif, exhibiting an even higher degree of sequence conservation compared to *E. coli*. Conservation of “G” or “C” at position “–4” was also uniformly observed in *P. aeruginosa* editing sites (Figure 3*A*). Taken together, these findings demonstrate that both pathogenic *E. coli* and *P. aeruginosa* display strong conservation of the 7-base editing motif, with the notable addition of conservation at position “−4,” which has not been previously reported.

We next asked whether these sequence motifs are preferentially embedded within loop regions of RNA secondary structures, as suggested by earlier studies [15, 18, 20]. Using MFE predictions with RNAfold [40], we found that, when considering local folding of 17 nucleotides flanking the edited site, 14 out of 23 sites in *E. coli* and 4 out of 7 in *P. aeruginosa* were situated in predicted loop structures (Figure 3*B* and Supplementary Figures 3–4). Recognizing the limitations of this local approach, we expanded the sequence window to 37 bases for sites not initially identified within loops. Using this extended context, nearly all remaining editing sites were also predicted to reside within loop structures, yielding a total of 20 out of 23 sites in *E. coli* and all seven sites in *P. aeruginosa* (Supplementary Figure 5).

In summary, our results demonstrate that in pathogenic *E. coli* and *P. aeruginosa*, A-to-I mRNA editing events predominantly occur within a conserved 7-base motif embedded in RNA loop structures, analogous to the configuration observed in the canonical edited site of *tRNA-Arg2*.

### Adenosine-to-inosine RNA Editing Occurs Regardless of the Isolate's Antimicrobial Resistance Profile, Patient Gender, Patient Age, Infection Severity, and Recurrence of Infection

We next sought to understand if editing occurrence or levels depends on the isolate or patient clinical parameters.

**Figure 4.**
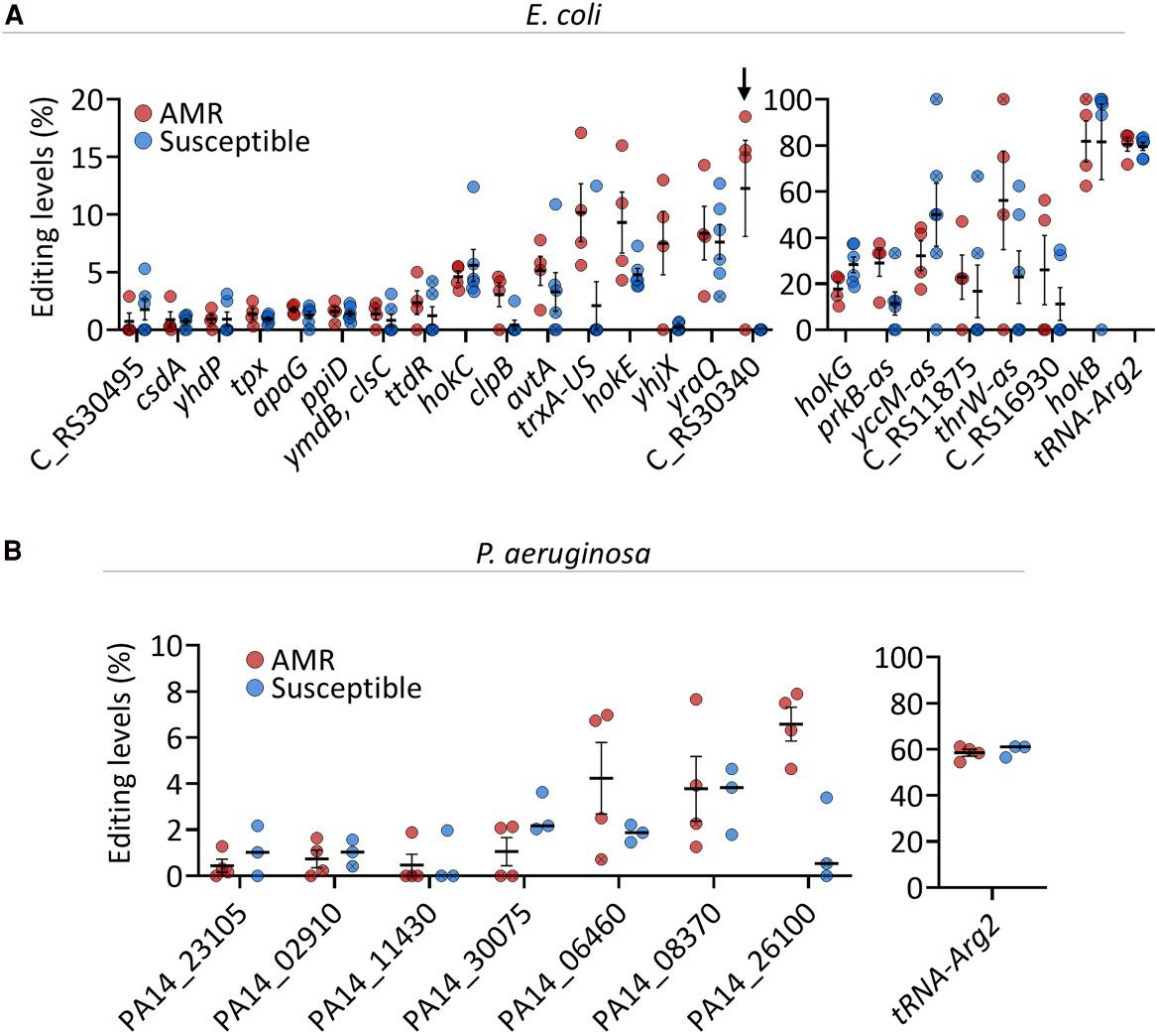
Adenosine-to-inosine RNA editing patterns among AMR and susceptible *E. coli* and *P. aeruginosa* clinical isolates. *A*, Adenosine-to-inosine RNA editing sites and levels in different genes in AMR and susceptible *E. coli* clinical isolates. A black arrow marks the only sites that were found in AMR (3/4) but not in any susceptible samples. *B*, Adenosine-to-inosine RNA editing sites and levels in different genes in AMR and susceptible *P. aeruginosa* clinical isolates. Student's or Welch's *t*-test was used to analyze all genes shown in A and B, followed by Benjamini–Hochberg false discovery rate correction (Supplementary Table 6). The difference in editing levels in all genes was not significant. Editing event/sites that do not match all the following criteria are marked with an “x”: a coverage of at least 4 reads, at least 2 reads supporting an event, with editing level above 1% (fraction of RNA reads with A-to-G mismatch, that are not found in the DNA data). All the data points presented here are found in Supplementary Tables 1 and 3.

To determine whether A-to-I RNA editing differs between AMR and susceptible isolates, we analyzed the occurrence and levels of editing in the different clinical isolates. Our *E. coli* dataset comprised four isolates resistant to beta-lactam and fluoroquinolone antibiotics and six isolates sensitive to all tested antibiotics (Supplementary Tables 5 and 6). Overall, we observed no significant differences in the number or levels of most A-to-I mRNA editing sites between resistant and susceptible groups (Figure 4*A* and Supplementary Table 7). An exception was identified at a site located four bases upstream of locus tag “CR_S30340,” which encodes an FadR C-terminal domain-containing protein. Editing at this site was detected exclusively in resistant isolates (3 out of 4), and not in susceptible isolates.

In *P. aeruginosa*, we examined four fluoroquinolone-resistant and three fluoroquinolone-sensitive isolates (Supplementary Tables 5 and 6). Here, as in *E. coli*, the same editing sites were present at comparable levels in both AMR and susceptible isolates (Figure 4*B* and Supplementary Table 6). Finally, to increase the power of our analysis for both *E. coli* and *P. aeruginosa*, we analyzed all editing events together for the susceptible and AMR isolates of each species. Still, no significant difference was observed between the susceptible and AMR isolates (Supplementary Figures 6 and 7).

Next, we sought to examine if editing levels differ between isolates based on patient and disease parameters. Examining editing levels according to patient age, gender, recurrence of infection, and infection severity did not reveal differences in mRNA editing levels between the isolates, in both *E. coli* and *P. aeruginosa* (Supplementary Figures 6 and 7 and Supplementary Tables 5–8).

Together, these findings indicate that, in general, both tRNA and mRNA A-to-I editing occur independently of AMR phenotypes or patients’ clinical parameters in the examined pathogenic *E. coli* and *P. aeruginosa* isolates.

## DISCUSSION

In this study, we characterized A-to-I RNA editing patterns in pathogenic bacteria isolated from hospitalized patients, focusing on *E. coli* and *P. aeruginosa*—common causative agents of UTI and otitis, respectively. Notably, our analyses revealed a significantly higher number and greater abundance of editing events in pathogenic *E. coli* isolates compared to laboratory strains [18]. Similarly, in *P. aeruginosa*, clinical isolates exhibited increased levels of RNA editing relative to what we previously observed in the reference PAO1 strain, in which only a single site was detected across 64 surveyed gammaproteobacterial species [17]. These findings underscore the possibility that both the occurrence and extent of A-to-I mRNA editing can vary substantially between strains within a species.

Our results demonstrate that mRNA editing is consistently more abundant in pathogenic *E. coli* than in *P. aeruginosa*, raising the intriguing possibility that A-to-I editing may play a more substantial biological role in *E. coli*. Alternatively, the limited number of sites observed in *P. aeruginosa* may reflect the culture conditions used in this study. All experiments were conducted in BHI broth, a standard clinical microbiology medium that does not fully replicate the physiological environments encountered by bacteria within the human host. RNA editing levels and patterns in *P. aeruginosa* may therefore differ under host-like conditions, such as within patient-derived fluids or tissue environments. Future studies should examine the occurrence and extent of RNA editing under more physiologically relevant conditions, for example, in human urine, serum, or infection-model systems.

What adaptive or pathogenic advantages can editing provide for pathogenic bacteria? RNA editing may provide several adaptive advantages for pathogenic bacteria by introducing functional diversity in a DNA-independent manner. When editing occurs within CDS, it can generate subpopulations expressing variant enzyme isoforms, thus creating phenotypic heterogeneity that may enhance adaptability under fluctuating environmental or host conditions. Most nonsynonymous editing events recode a tyrosine to a cysteine codon. Subsequently, editing may regulate disulfide bond formation in edited proteins, as we recently showed in the case of HokB [21].

RNA Editing can also contribute to transcript-level variability without changing protein sequence—for instance, through synonymous substitutions (eg*, yhdP*), modifications within untranslated regions (eg*, trxA 5*′ *UTR*), or in transcripts transcribed from the AS strand of known genes (eg*, thrW*-as). Such noncoding editing events could influence translation initiation or disrupt small RNA binding sites, thereby modulating RNA stability or post-transcriptional regulation. These mechanisms may confer selective advantages by fine-tuning gene expression or enzymatic activity, enabling bacterial pathogens to adjust to stress, immune pressure, or nutrient limitations encountered in the human host environment.

Here, editing profiles did not differ significantly between antibiotic-resistant and susceptible isolates of *E. coli* and *P. aeruginosa*. Thus, at least under our growth conditions and experimental design, A-to-I RNA editing does not directly contribute to AMR, which is likely caused by known genes and mutations that mediate AMR (Supplementary Table 6). Nonetheless, its broader roles in bacterial physiology and environmental adaptation remain an important avenue for future investigation.

Notably, not all editing events necessarily have a functional role. We cannot exclude the possibility that some of the observed editing events, particularly those detected at low levels, represent off-target activity of the TadA enzyme rather than biologically meaningful modifications. This consideration is especially relevant in *P. aeruginosa* clinical isolates, where all identified mRNA editing events occurred at consistently low frequencies (<8%). While such low-level events may still contribute to subtle functional diversification or population heterogeneity, distinguishing regulated editing from incidental enzymatic activity will require targeted validation and physiological context-dependent assays.

To elucidate the functional impact of specific editing events, targeted gene-editing approaches will be necessary. Specifically, construction of mutants lacking editing at defined sites, followed by assessment of their growth and pathogenicity in clinically relevant models, will help determine whether mRNA editing influences bacterial fitness or virulence during infection.

Consistent with our previous findings in gammaproteobacteria [17], we found that editing events in both pathogenic *E. coli* and *P. aeruginosa* predominantly occur within a conserved 7-base motif. Interestingly, this motif appears more flexible in *E. coli* but is highly conserved in *P. aeruginosa*, suggesting that TadA exhibits stricter substrate specificity in the latter. These differences imply that the sequence recognition and editing preferences of TadA may have adapted in a species-specific manner.

In summary, our work provides evidence that A-to-I mRNA editing is a feature of pathogenic *E. coli* and *P. aeruginosa* isolates. These findings establish a foundation for future studies aimed at mapping editing landscapes across additional clinical isolates and infection-relevant conditions, and at uncovering the functional consequences of mRNA editing for bacterial pathogenesis and interaction with the host.

## Supplementary Material

jiaf645_Supplementary_Data

## Supplementary Data

Supplementary materials are available at *The Journal of Infectious Diseases* online (http://jid.oxfordjournals.org/). Supplementary materials consist of data provided by the author that are published to benefit the reader. The posted materials are not copyedited. The contents of all supplementary data are the sole responsibility of the authors. Questions or messages regarding errors should be addressed to the author.
