# Supplementary material for: The Landscape and Regulatory Determinants of A-to-I RNA Editing in Escherichia coli and Pseudomonas aeruginosa Isolated From Patients With Urinary Tract and Ear Infections
Source: J Infect Dis. 2025 Dec 24;233(3):e721–30. doi: 10.1093/infdis/jiaf645 (PMC13017025; doi:10.1093/infdis/jiaf645)
Supplement: jiaf645_Supplementary_Data [file jiaf645_supplementary_data.zip › Elias_et_al_JID-83483_Supplementary_Material_Clean.docx]

**The landscape and regulatory determinants of A-to-I RNA editing in *Escherichia coli* and *Pseudomonas aeruginosa* isolated from patients with urinary tract and ear infections**

Eyal Elias^1^, Danielle Keidar-Friedman^2^, Nadav Sorek^2^, Orit Raz^2^, Sharon Ovnat Tamir^2^, Liam Aspit^1^, and Dan Bar Yaacov^1*^

^1^The Shraga Segal Department of Microbiology, Immunology, and Genetics, Ben-Gurion University of the Negev, Israel; ^2^Assuta Ashdod University Hospital, Faculty of Health Sciences, Ben Gurion University of the Negev.

*Correspondence: danbary@bgu.ac.il

**Supplementary Figures and Supplementary Tables’ legends**

**Supplementary Figures**

**
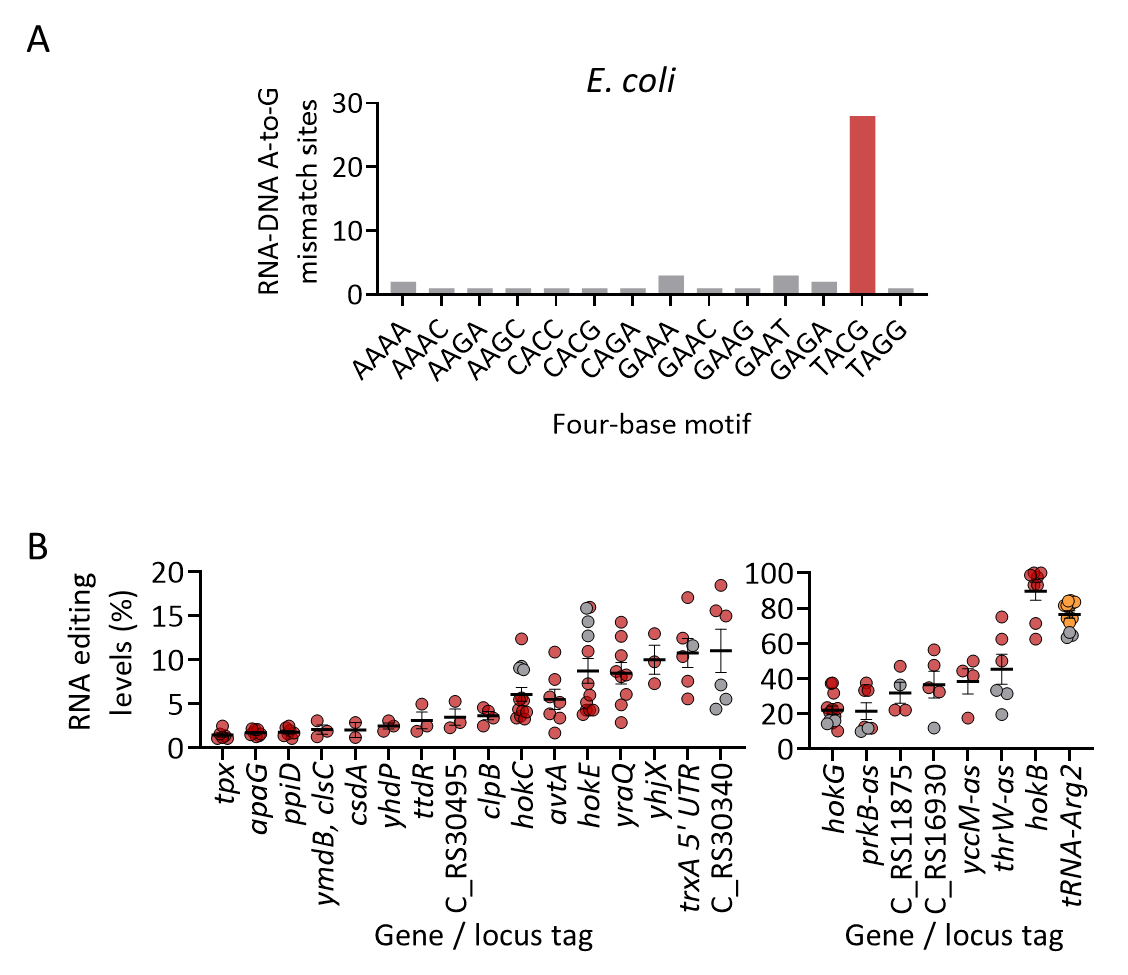
**

**Supplementary Figure 1. Editing Sequence motif and levels in clinical isolates and uropathogenic *E. coli* reference strain (CFT073). A.** Sequence distribution around A-to-G RNA-DNA mismatches in their genomic context as identified in the analyzed *E. coli* clinical isolates . **B.** An overview of RNA editing levels in identified transcripts in the clinical isolates described in Figure 1G and in the UPEC reference strain (CFT073 – in grey). Each grey dot represents one of three available samples of the CFT073 strain. In some sites, we did not observe editing at all, while in others, we observed editing in one to three samples.

As shown in Figure 1G, the left panel displays sites with an average editing rate below 20%. On the right panel are sites with an average editing of above 20%. Only sites that passed our filters in at least two samples are displayed (see Materials and Methods). In unannotated genes, the locus tag is mentioned. Each editing event shown here must have a coverage of at least 4 reads, at least 2 reads supporting an event, with editing level above 1% (fraction of RNA reads with A-to-G mismatch, that are not found in the DNA data), and present in at least 2 samples (isolates).

**
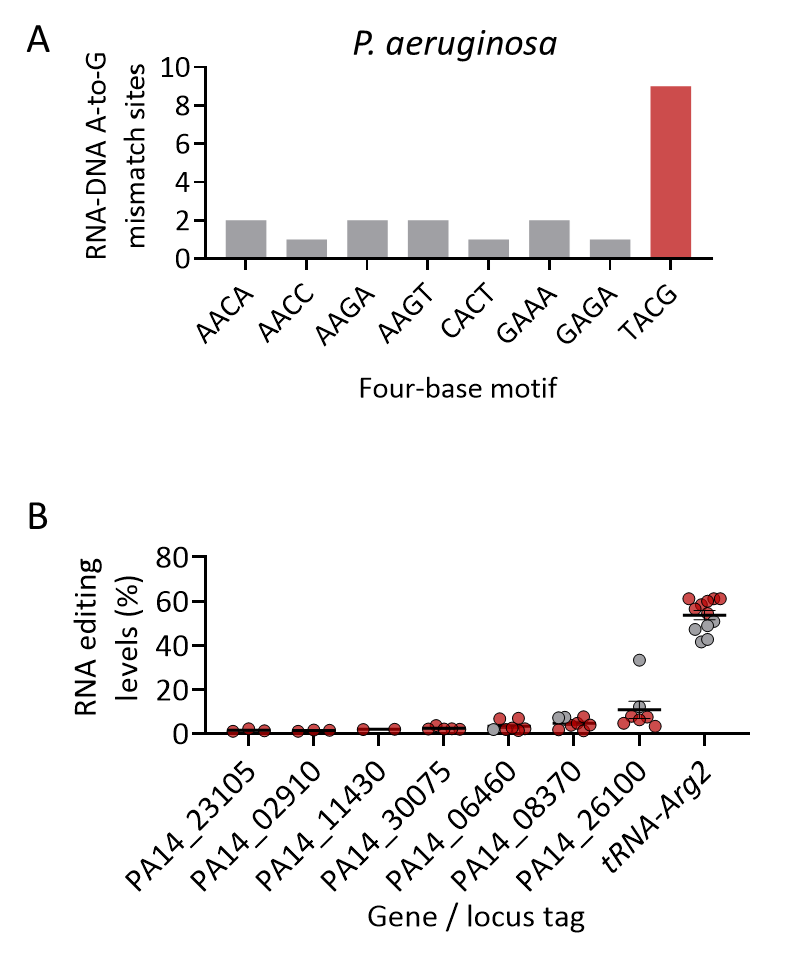
**

**Supplementary Figure 2. Editing Sequence motif and levels in clinical isolates and pathogenic *P.* *aeruginosa* reference strain (UCBPP-PA14). A.** Sequence distribution around A-to-G RNA-DNA mismatches in their genomic context as identified in the analyzed *P. aeruginosa* clinical isolates. **B.** An overview of RNA editing levels in identified transcripts in the clinical isolates described in Figure 1G and in the *P. aeruginosa* reference strain (UCBPP-PA14 – in grey). Each grey dot represents one of five available samples of the UCBPP-PA14 strain. In some sites, we did not observe editing at all, while in others, we observed editing in one to five samples.

As shown in Figure 1G, the left panel displays sites with an average editing rate below 20%. On the right panel are sites with an average editing of above 20%. Only sites that passed our filters in at least two samples are displayed (see Materials and Methods). In unannotated genes, the locus tag is mentioned. Each editing event shown here must have a coverage of at least 4 reads, at least 2 reads supporting an event, with editing level above 1% (fraction of RNA reads with A-to-G mismatch, that are not found in the DNA data), and present in at least 2 samples (isolates).

**
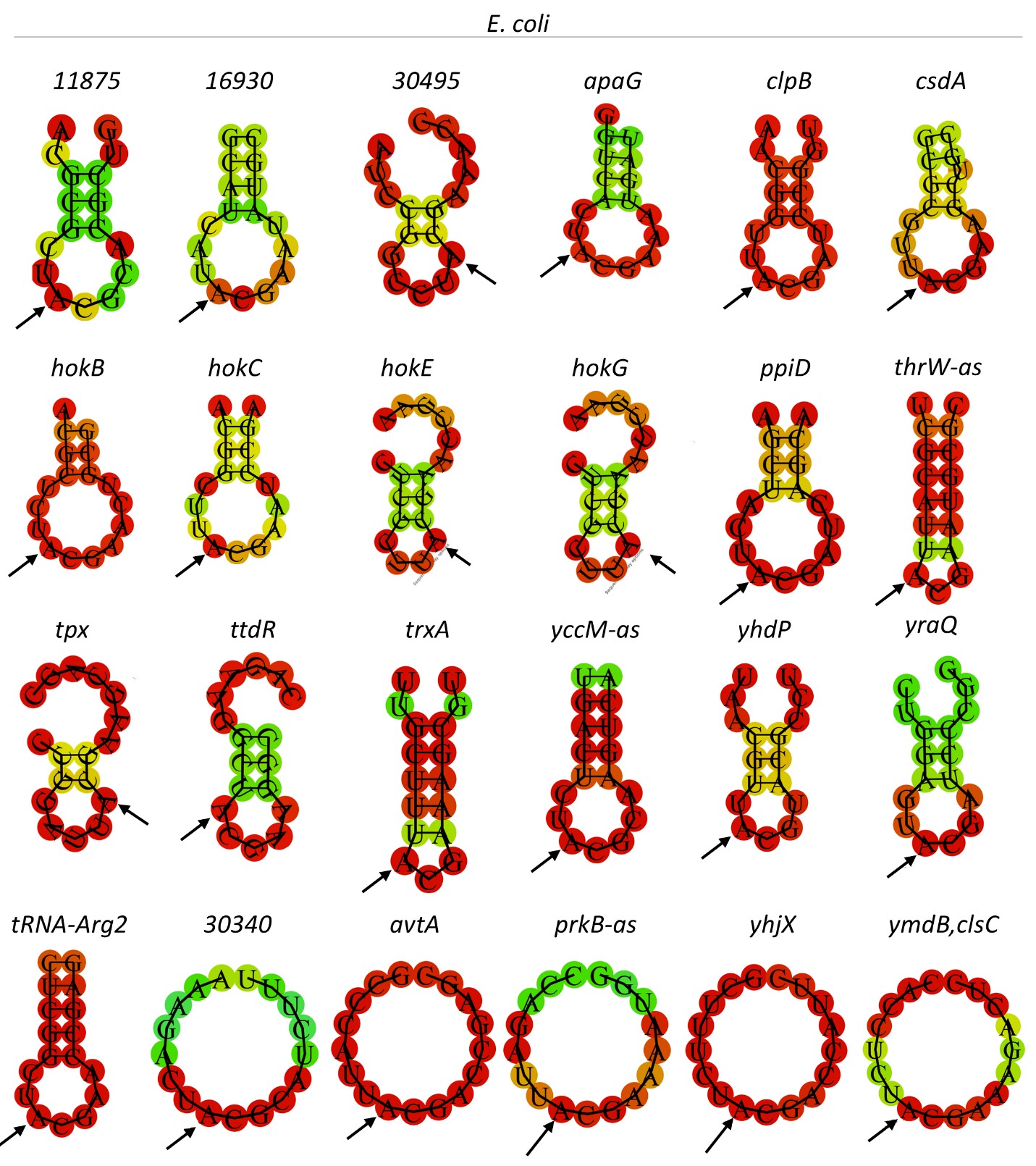
**

**Supplementary Figure 3.** Minimum free energy (MFE) secondary structure predicted by RNAfold around A-to-I editing sites (marked by a black arrowhead). Predictions were made using 17 nucleotides around the edited site in all 23 edited mRNAs and *tRNA-Arg2* of *E. coli* as original output from RNAfold.

**
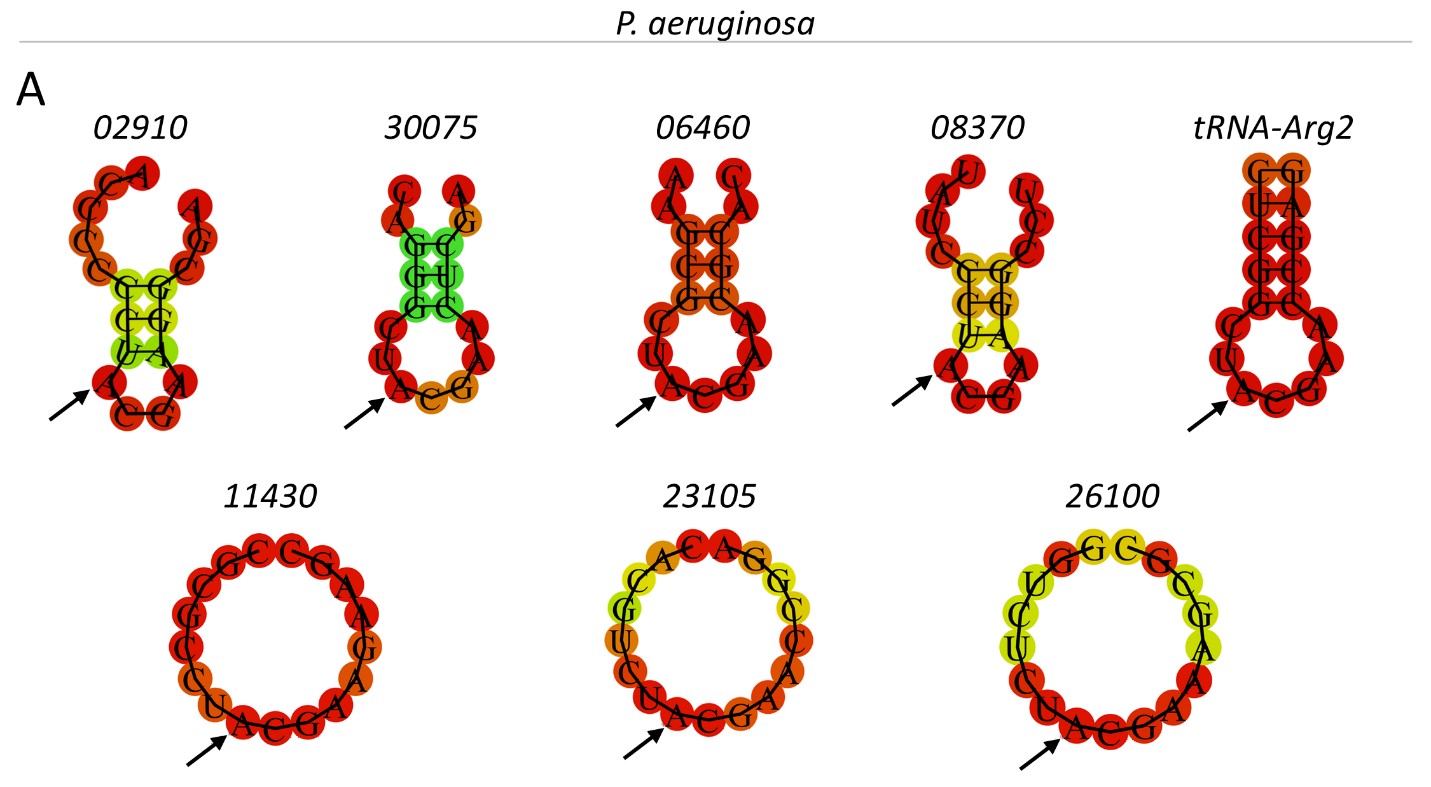
**

**Supplementary Figure 4.** Minimum free energy (MFE) secondary structure predicted by RNAfold around A-to-I editing sites (marked by a black arrow head). Predictions were made using 17 nucleotides around the edited site in all seven edited mRNAs and *tRNA-Arg2* of *P. aeruginosa* as originally output from RNAfold.

**
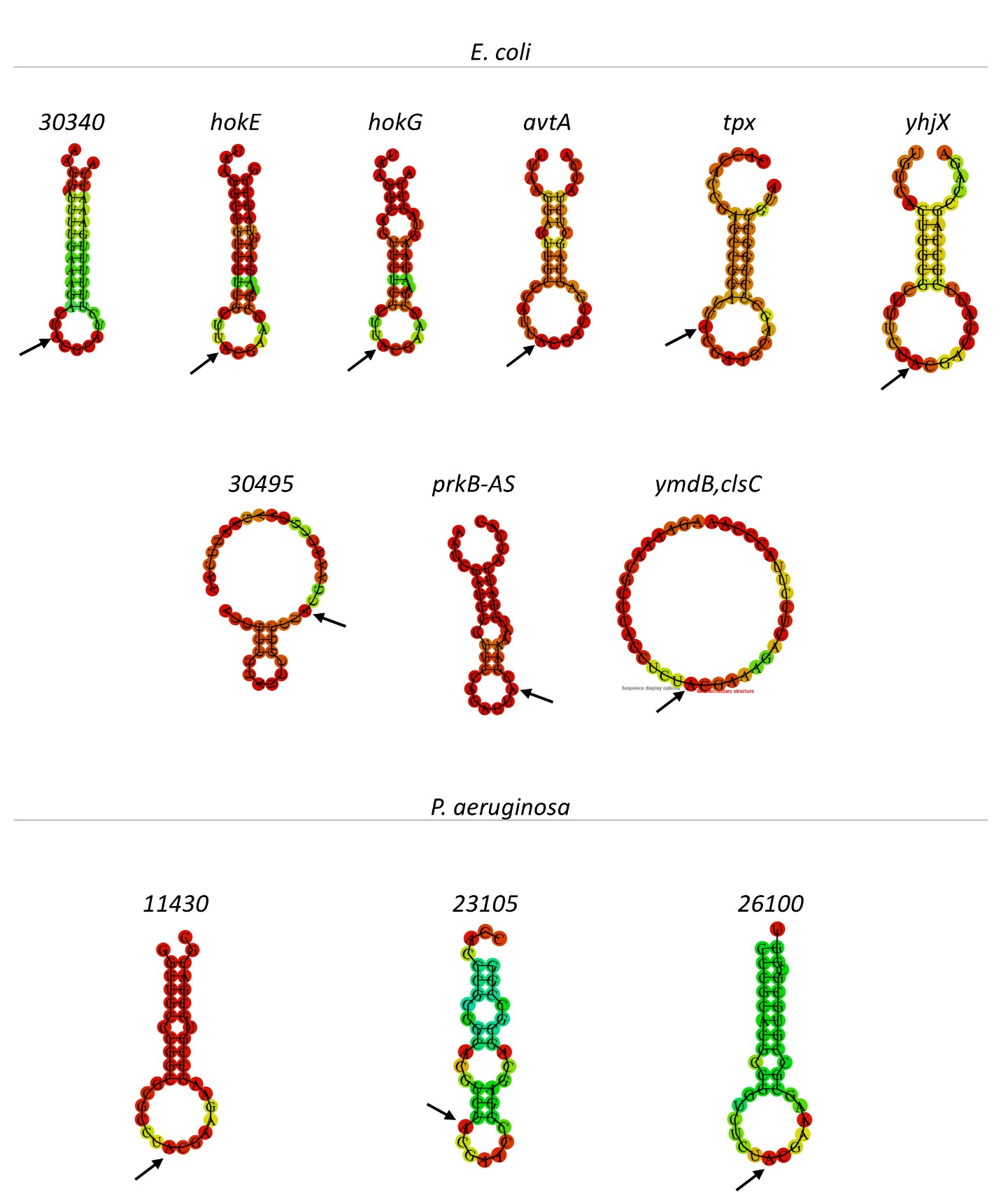
**

**Supplementary Figure 5.** Minimum free energy (MFE) secondary structure predicted by RNAfold around A-to-I editing sites (marked by a black arrow head). Predictions were made using 37 nucleotides around the edited site in mRNA edited sites that were not located to a loop using 17 nucleotides around the edited site. Shown is the original output from RNAfold.

**
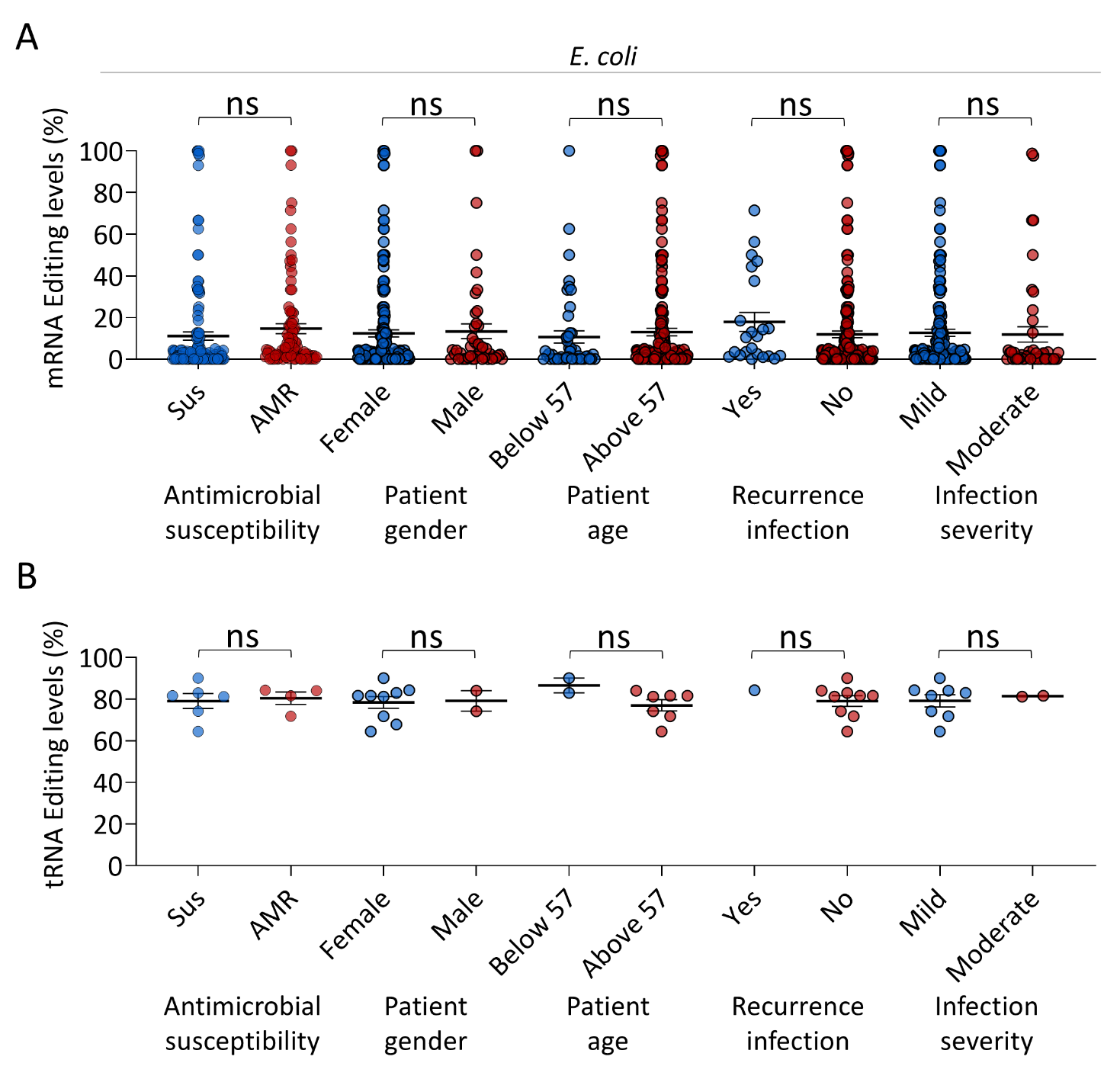
**

**Supplementary Figure 6. A-to-I RNA editing levels in *E. coli* clinical isolates according to isolates and patient clinical parameters.** Clinical parameters are listed in Supplementary Tables 5 – 8. Student's *t*-test was used to analyze all pairwise comparisons. Notably, for patient age, we only used adults (>18) and decided to categorize people as either above 57 or below 57 based on age distribution of the patients.

**
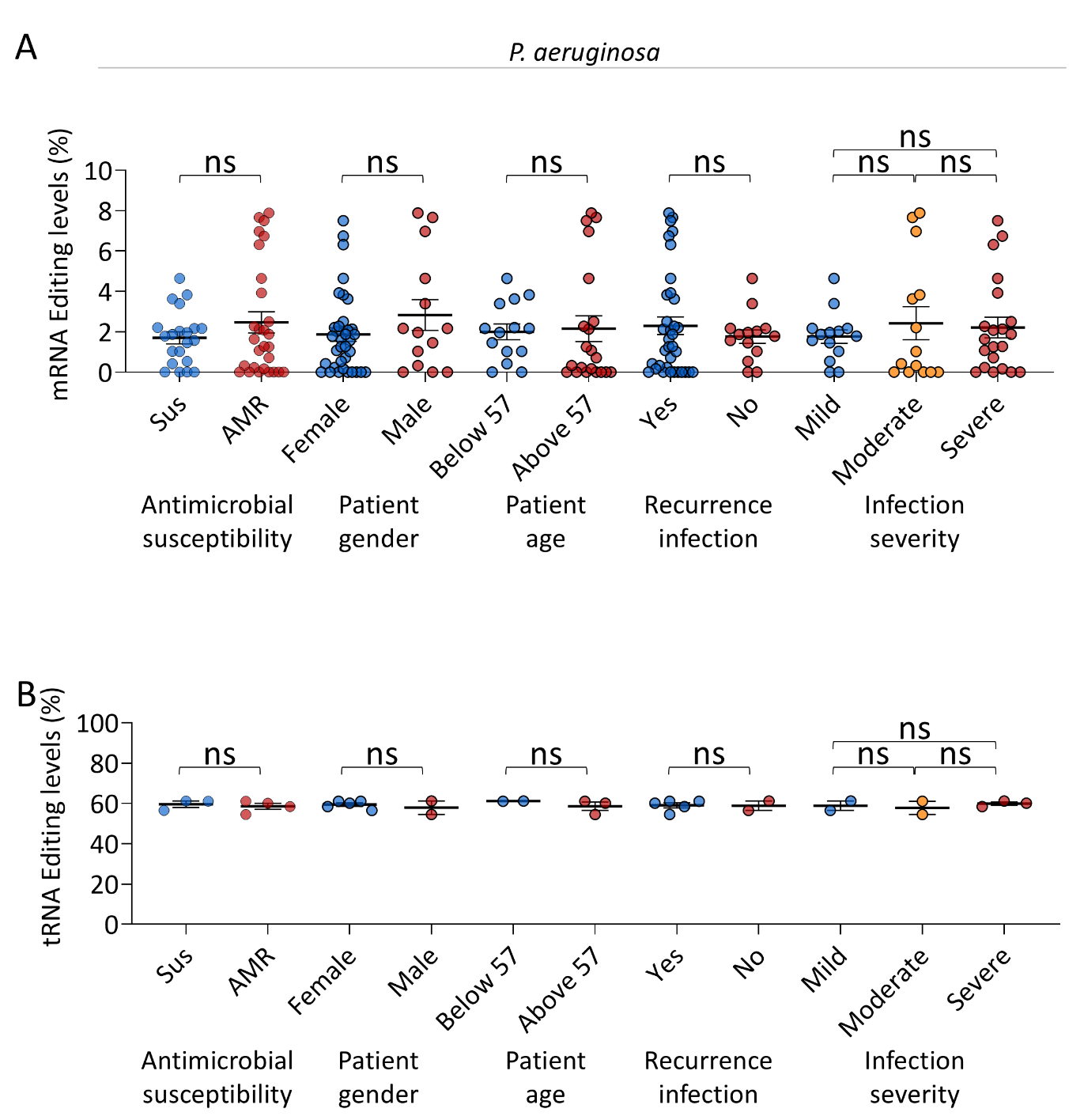
Supplementary Figure 7. A-to-I RNA editing levels in *P. aeruginosa* clinical isolates according to isolates and patient clinical parameters.** Clinical parameters are found in Supplementary Tables 5 – 8. Student's *t*-test was used to test pairwise comparisons, and one-way ANOVA was used to test editing levels between disease severity categories. Notably, for patient age, we only used adults (>18) and decided to categorize people as either above 57 or below 57 based on age distribution of the patients.

**Supplementary Tables Legend**

**Supplementary Table 1. RNA editing events in *E. coli* isolated from patients with urinary tract infection.** In black text, editing events that passed our filters and are shown in Figure 1. For an editing event to be present it has to have a coverage of at least 4 reads, at least 2 reads supporting an event, with editing level above 1%, and present in at least 2 samples. In grey text, editing events that did not pass our filters and were extracted from the mapping file.

**Supplementary Table 2. RNA editing events in *E. coli* reference strain CFT073.** Shown are all editing events that passed our filters as explained in **Supplementary Table 1** and are shown in Supplementary Figure 2.

**Supplementary Table 3. RNA editing events in *P. aeruginosa* isolated from patients with ear infection.** In black text, editing events that passed our filters as explained in **Supplementary Table 1** and are shown in Figure 2. In grey text, editing events that did not pass our filters and were extracted from the mapping file.

**Supplementary Table 4. RNA editing events in *P. aeruginosa* reference strain UCBPP-PA14.** Shown are all editing events that passed our filters as explained in **Supplementary Table 1** and are shown in Supplementary Figure 2.

**Supplementary Table 5.** Antibiogram of the different bacterial isolates that were used in the research. “EC”; isolates of *E. coli*, “PA”; isolates of *P. aeruginosa*, “S”; sensitive, “I”; intermediate, “R”; resistant.

**Supplementary Table 6.** Genes and mutations known to confer antimicrobial resistance in the examined species.

**Supplementary Table 7.** Statistical analysis to examine differences in editing levels between AMR and susceptible isolates of *E. coli* and *P. aeruginosa*.

**Supplementary Table 8.** Patients’ clinical parameters of each isolate analyzed in this study.

**Supplementary Table 9.** List of primers that were used in PCR reactions for A-to-I mRNA editing sites validations in the UPEC clinical isolates using Sanger sequencing.
